# Crystal structure of bis­(2,2′-bi­pyridine-κ^2^ *N*,*N*′)bis­(thio­cyanato-κ*N*)mang­anese(II) 2,2′-bi­pyridine monosolvate

**DOI:** 10.1107/S205698901402516X

**Published:** 2015-01-01

**Authors:** Stefan Suckert, Inke Jess, Christian Näther

**Affiliations:** a Institut für Anorganische Chemie, Christian-Albrechts-Universität Kiel, Max-Eyth-Strasse 2, 24118 Kiel, Germany

**Keywords:** crystal structure, coordination polymer, octa­hedral manganese(II) coordination

## Abstract

In the crystal structure of the mononuclear title compound, [Mn(NCS)_2_(C_10_H_8_N_2_)_2_]·C_10_H_8_N_2_, the Mn^II^ cation is coordin­ated in an all-*cis* configuration by two N-bound thio­cyanate anions and two 2,2′-bi­pyridine ligands within a slightly distorted octa­hedral environment. The asymmetric unit consists of one Mn^II^ cation, two thio­cyanate anions and two 2,2′-bi­pyridine ligands, as well as two non-coordinating 2,2′-bi­pyridine ligands that are each located on centres of inversion. In the crystal structure, the discrete [Mn(NCS)_2_(C_10_H_8_N_2_)_2_] complex mol­ecules are arranged in such a way that cavities are formed, in which the solvent 2,2′-bi­pyridine mol­ecules are located. Apart from van der Waals forces, there are no remarkable inter­molecular inter­actions present in the crystal structure.

## Related literature   

For similar crystal structures with thio­cyanate anions in *cis*-coordination to a manganese(II) cation, see: Małecki *et al.* (2011 ▸).
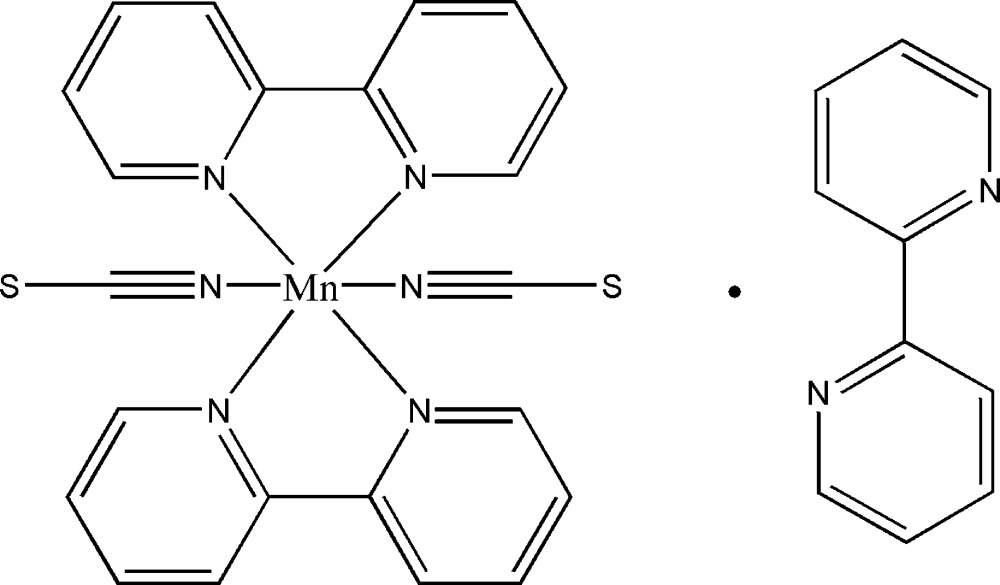


## Experimental   

### Crystal data  

[Mn(NCS)_2_(C_10_H_8_N_2_)_2_]·C_10_H_8_N_2_
*M* *_r_* = 639.65
Monoclinic, 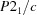

*a* = 14.5263 (6) Å
*b* = 13.5383 (4) Å
*c* = 16.0726 (7) Å
β = 105.535 (3)°
*V* = 3045.4 (2) Å^3^
*Z* = 4
Mo *K*α radiation
μ = 0.61 mm^−1^
*T* = 293 K
0.32 × 0.19 × 0.07 mm

### Data collection  

Stoe IPDS-2 diffractometer
Absorption correction: numerical (*X-SHAPE* and *X-RED32*; Stoe & Cie, 2008 ▸) *T*_min_ = 0.820, *T*_max_ = 0.919
41565 measured reflections
6630 independent reflections
5766 reflections with *I* > 2σ(*I*)
*R*_int_ = 0.037

### Refinement  

*R*[*F* ^2^ > 2σ(*F* ^2^)] = 0.041
*wR*(*F* ^2^) = 0.094
*S* = 1.08
6630 reflections
389 parameters
H-atom parameters constrained
Δρ_max_ = 0.27 e Å^−3^
Δρ_min_ = −0.35 e Å^−3^

### 

Data collection: *X-AREA* (Stoe & Cie, 2008 ▸); cell refinement: *X-AREA*; data reduction: *X-AREA*; program(s) used to solve structure: *SHELXS97* (Sheldrick, 2008 ▸); program(s) used to refine structure: *SHELXL97* (Sheldrick, 2008 ▸); molecular graphics: *XP* in *SHELXTL* (Sheldrick, 2008 ▸) and *DIAMOND* (Brandenburg, 1999 ▸); software used to prepare material for publication: *publCIF* (Westrip, 2010 ▸).

## Supplementary Material

Crystal structure: contains datablock(s) I, global. DOI: 10.1107/S205698901402516X/wm5090sup1.cif

Structure factors: contains datablock(s) I. DOI: 10.1107/S205698901402516X/wm5090Isup2.hkl

**Fig. 1.**
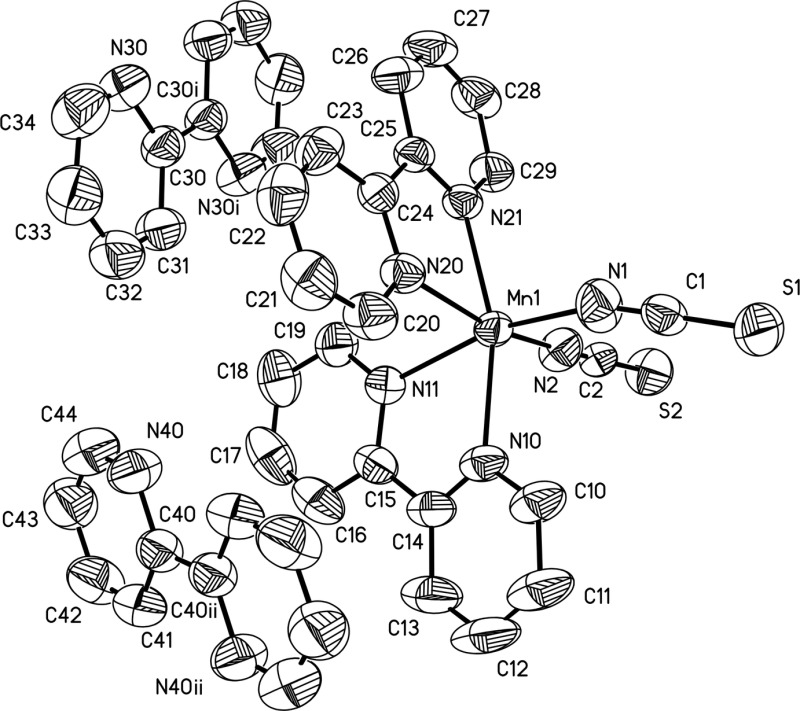
The molecular components of the title compound with labelling and displacement ellipsoids drawn at the 50% probability level. [Symmetry codes: (i) -x+1,-y+1,-z+1; (ii) -x+2,-y+1,-z+1.]

. DOI: 10.1107/S205698901402516X/wm5090fig1.tif
The mol­ecular components of the title compound with labelling and displacement ellipsoids drawn at the 50% probability level. [Symmetry codes: (i) −x+1,-y+1,-z+1; (ii) −x+2,-y+1,-z+1.]

**Fig. 2.**
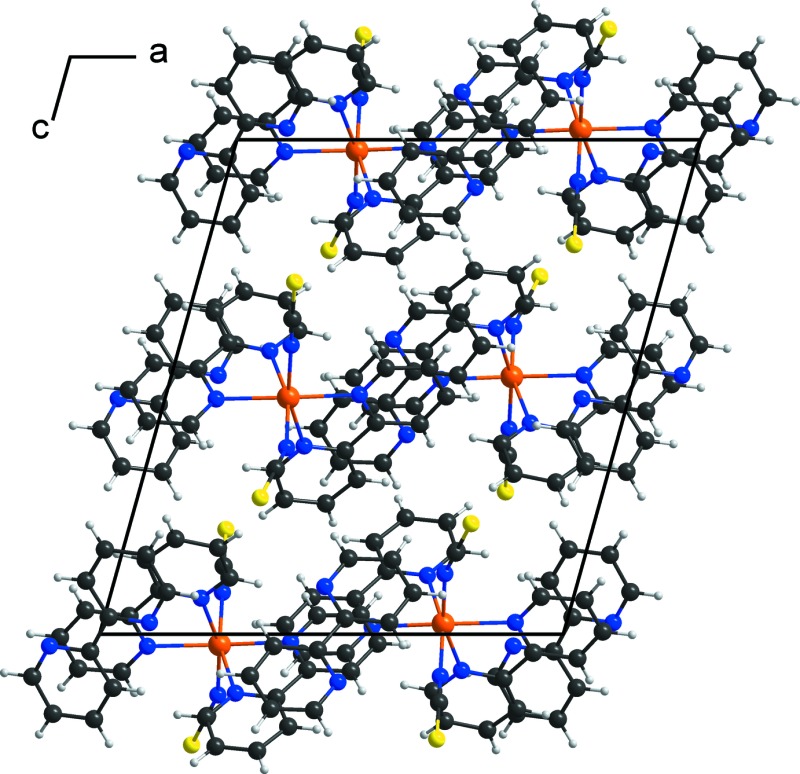
The packing of the molecules in the crystal structure of the title compound in a view along [010].

. DOI: 10.1107/S205698901402516X/wm5090fig2.tif
The packing of the mol­ecules in the crystal structure of the title compound in a view along [010].

CCDC reference: 1034429

Additional supporting information:  crystallographic information; 3D view; checkCIF report

## supplementary crystallographic information

### S1. Synthesis and crystallization

MnSO_4_·H_2_O was purchased from Merck and 2,2'-bi­pyridine and Ba(NCS)_2_·3H_2_O were purchased from Alfa Aesar. Mn(NCS)_2_ was synthesized by stirring 17.97 g (58.44 mmol) Ba(NCS)_2_·3H_2_O and 9.88 g (58.44 mmol) MnSO_4_·H_2_O in 300 ml water at room temperature for three hours. The white residue of BaSO_4_ was filtered off and the solvent removed with a rotary evaporator. The homogeneity of the product was investigated by X-ray powder diffraction and elemental analysis. The title compound was prepared by the reaction of 25.7 mg (0.15 mmol) Mn(NCS)_2_ and 93.7 mg (0.60 mmol) 2,2'-bi­pyridine in 1.0 ml water at room temperature. After few days, yellow plates of the title compound were obtained.

### S2. Refinement

The hydrogen atoms were positioned with idealized geometry and were refined with C—H = 0.93 Å and *U*_eq_(H) = 1.2 *U*_eq_(C) using a riding model approximation.

### Crystal data

**Table:**

[Mn(NCS)_2_(C_10_H_8_N_2_)_2_]·C_10_H_8_N_2_	*Z* = 4
*M**_r_* = 639.65	*F*(000) = 1316
Monoclinic, *P*2_1_/*c*	*D*_x_ = 1.395 Mg m^−^^3^
Hall symbol: -P 2ybc	Mo *K*α radiation, λ = 0.71073 Å
*a* = 14.5263 (6) Å	θ = 1.5–27.0°
*b* = 13.5383 (4) Å	µ = 0.61 mm^−^^1^
*c* = 16.0726 (7) Å	*T* = 293 K
β = 105.535 (3)°	Plate, yellow
*V* = 3045.4 (2) Å^3^	0.32 × 0.19 × 0.07 mm

### Data collection

**Table:**

Stoe IPDS-2 diffractometer	6630 independent reflections
Radiation source: fine-focus sealed tube	5766 reflections with *I* > 2σ(*I*)
Graphite monochromator	*R*_int_ = 0.037
ω scans	θ_max_ = 27.0°, θ_min_ = 1.5°
Absorption correction: numerical (*X-SHAPE* and *X-RED32*; Stoe & Cie, 2008)	*h* = −18→18
*T*_min_ = 0.820, *T*_max_ = 0.919	*k* = −17→17
41565 measured reflections	*l* = −20→20

### Refinement

**Table:**

Refinement on *F*^2^	Secondary atom site location: difference Fourier map
Least-squares matrix: full	Hydrogen site location: inferred from neighbouring sites
*R*[*F*^2^ > 2σ(*F*^2^)] = 0.041	H-atom parameters constrained
*wR*(*F*^2^) = 0.094	*w* = 1/[σ^2^(*F*_o_^2^) + (0.0384*P*)^2^ + 0.9654*P*] where *P* = (*F*_o_^2^ + 2*F*_c_^2^)/3
*S* = 1.08	(Δ/σ)_max_ = 0.001
6630 reflections	Δρ_max_ = 0.27 e Å^−^^3^
389 parameters	Δρ_min_ = −0.35 e Å^−^^3^
0 restraints	Extinction correction: *SHELXL97* (Sheldrick, 2008), Fc^*^=kFc[1+0.001xFc^2^λ^3^/sin(2θ)]^-1/4^
Primary atom site location: structure-invariant direct methods	Extinction coefficient: 0.0025 (9)

### Special details

**Table:**

Geometry. All esds (except the esd in the dihedral angle between two l.s. planes) are estimated using the full covariance matrix. The cell esds are taken into account individually in the estimation of esds in distances, angles and torsion angles; correlations between esds in cell parameters are only used when they are defined by crystal symmetry. An approximate (isotropic) treatment of cell esds is used for estimating esds involving l.s. planes.
Refinement. Refinement of F^2^ against ALL reflections. The weighted R-factor wR and goodness of fit S are based on F^2^, conventional R-factors R are based on F, with F set to zero for negative F^2^. The threshold expression of F^2^ > 2sigma(F^2^) is used only for calculating R-factors(gt) etc. and is not relevant to the choice of reflections for refinement. R-factors based on F^2^ are statistically about twice as large as those based on F, and R- factors based on ALL data will be even larger.

### Fractional atomic coordinates and isotropic or equivalent isotropic displacement parameters (Å^2^)

**Table:**

	*x*	*y*	*z*	*U*_iso_*/*U*_eq_	
Mn1	0.734793 (17)	0.128336 (19)	0.478914 (17)	0.04106 (9)	
N1	0.70830 (14)	0.02009 (14)	0.37737 (12)	0.0665 (5)	
C1	0.71894 (13)	−0.04547 (14)	0.33491 (12)	0.0502 (4)	
S1	0.73507 (5)	−0.13676 (5)	0.27623 (4)	0.0792 (2)	
N2	0.75999 (12)	0.02533 (13)	0.58481 (11)	0.0583 (4)	
C2	0.77308 (12)	−0.03438 (13)	0.63794 (12)	0.0453 (4)	
S2	0.79008 (5)	−0.11880 (5)	0.71196 (4)	0.07089 (17)	
N10	0.88949 (10)	0.14680 (12)	0.47586 (10)	0.0513 (4)	
C10	0.92943 (15)	0.09366 (18)	0.42424 (15)	0.0652 (6)	
H10	0.8898	0.0546	0.3818	0.078*	
C11	1.02539 (17)	0.0938 (2)	0.43073 (19)	0.0824 (8)	
H11	1.0502	0.0572	0.3928	0.099*	
C12	1.08341 (17)	0.1492 (2)	0.4943 (2)	0.0908 (9)	
H12	1.1490	0.1497	0.5010	0.109*	
C13	1.04482 (15)	0.2043 (2)	0.54863 (18)	0.0792 (7)	
H13	1.0840	0.2420	0.5924	0.095*	
C14	0.94611 (13)	0.20298 (15)	0.53718 (13)	0.0541 (5)	
C15	0.89768 (13)	0.26359 (14)	0.58963 (12)	0.0517 (4)	
C16	0.94487 (18)	0.33141 (19)	0.65064 (15)	0.0728 (6)	
H16	1.0108	0.3385	0.6629	0.087*	
C17	0.8933 (2)	0.3882 (2)	0.69288 (16)	0.0852 (8)	
H17	0.9244	0.4343	0.7337	0.102*	
C18	0.7964 (2)	0.37728 (17)	0.67522 (16)	0.0771 (7)	
H18	0.7605	0.4157	0.7030	0.092*	
C19	0.75380 (16)	0.30723 (16)	0.61475 (13)	0.0607 (5)	
H19	0.6880	0.2987	0.6025	0.073*	
N11	0.80238 (10)	0.25125 (11)	0.57302 (10)	0.0488 (3)	
N20	0.67171 (10)	0.24892 (11)	0.38090 (9)	0.0470 (3)	
C20	0.72036 (15)	0.29317 (17)	0.33176 (14)	0.0624 (5)	
H20	0.7831	0.2736	0.3374	0.075*	
C21	0.68249 (19)	0.36615 (18)	0.27327 (15)	0.0726 (6)	
H21	0.7188	0.3955	0.2404	0.087*	
C22	0.59047 (19)	0.39454 (17)	0.26467 (14)	0.0713 (6)	
H22	0.5632	0.4443	0.2260	0.086*	
C24	0.58074 (12)	0.27636 (13)	0.37188 (11)	0.0446 (4)	
C25	0.53069 (12)	0.22504 (13)	0.42834 (11)	0.0438 (4)	
C26	0.43568 (13)	0.24294 (17)	0.42531 (14)	0.0613 (5)	
H26	0.4000	0.2878	0.3858	0.074*	
C27	0.39498 (14)	0.19383 (19)	0.48122 (15)	0.0689 (6)	
H27	0.3314	0.2050	0.4797	0.083*	
C28	0.44834 (15)	0.12849 (17)	0.53914 (15)	0.0652 (6)	
H28	0.4223	0.0955	0.5782	0.078*	
C29	0.54214 (14)	0.11259 (15)	0.53824 (13)	0.0538 (4)	
H29	0.5782	0.0671	0.5768	0.065*	
N21	0.58328 (10)	0.15956 (11)	0.48459 (9)	0.0427 (3)	
N30	0.45727 (13)	0.57292 (15)	0.40356 (13)	0.0690 (5)	
C30	0.52000 (14)	0.52678 (15)	0.46832 (13)	0.0557 (5)	
C31	0.61789 (15)	0.52953 (17)	0.47611 (15)	0.0654 (5)	
H31	0.6605	0.4974	0.5216	0.078*	
C23	0.53780 (16)	0.34899 (15)	0.31364 (13)	0.0597 (5)	
H23	0.4745	0.3668	0.3076	0.072*	
C32	0.65066 (18)	0.5801 (2)	0.41603 (18)	0.0766 (6)	
H32	0.7158	0.5826	0.4203	0.092*	
C33	0.5866 (2)	0.6271 (2)	0.34934 (19)	0.0813 (7)	
H33	0.6069	0.6617	0.3075	0.098*	
C34	0.4917 (2)	0.6212 (2)	0.34664 (18)	0.0822 (7)	
H34	0.4483	0.6533	0.3017	0.099*	
N40	0.89931 (14)	0.56056 (16)	0.52370 (14)	0.0753 (5)	
C40	0.99023 (15)	0.53074 (16)	0.53470 (14)	0.0617 (5)	
C41	1.06105 (17)	0.55483 (19)	0.60838 (17)	0.0756 (6)	
H41	1.1234	0.5332	0.6150	0.091*	
C42	1.0391 (2)	0.6106 (2)	0.67145 (19)	0.0877 (8)	
H42	1.0863	0.6269	0.7212	0.105*	
C43	0.9471 (2)	0.6423 (2)	0.6607 (2)	0.0887 (8)	
H43	0.9301	0.6808	0.7022	0.106*	
C44	0.8805 (2)	0.6148 (2)	0.5856 (2)	0.0891 (8)	
H44	0.8179	0.6360	0.5779	0.107*	

### Atomic displacement parameters (Å^2^)

**Table:**

	*U*^11^	*U*^22^	*U*^33^	*U*^12^	*U*^13^	*U*^23^
Mn1	0.03601 (14)	0.04340 (15)	0.04746 (15)	0.00108 (10)	0.01753 (10)	0.00063 (11)
N1	0.0761 (12)	0.0621 (10)	0.0701 (11)	−0.0069 (9)	0.0350 (9)	−0.0150 (9)
C1	0.0426 (9)	0.0562 (11)	0.0537 (10)	−0.0084 (8)	0.0160 (8)	−0.0048 (9)
S1	0.0676 (4)	0.0790 (4)	0.0828 (4)	0.0061 (3)	0.0057 (3)	−0.0377 (3)
N2	0.0596 (10)	0.0556 (9)	0.0651 (10)	0.0078 (8)	0.0263 (8)	0.0123 (8)
C2	0.0384 (9)	0.0475 (9)	0.0520 (10)	0.0034 (7)	0.0156 (7)	−0.0024 (8)
S2	0.0751 (4)	0.0693 (4)	0.0641 (3)	0.0054 (3)	0.0114 (3)	0.0214 (3)
N10	0.0365 (7)	0.0616 (9)	0.0595 (9)	0.0044 (7)	0.0193 (7)	0.0112 (7)
C10	0.0494 (11)	0.0803 (14)	0.0749 (14)	0.0122 (10)	0.0322 (10)	0.0105 (11)
C11	0.0504 (13)	0.110 (2)	0.0989 (19)	0.0219 (13)	0.0401 (13)	0.0237 (16)
C12	0.0393 (12)	0.122 (2)	0.118 (2)	0.0176 (13)	0.0336 (14)	0.0357 (19)
C13	0.0369 (10)	0.1010 (19)	0.0934 (17)	−0.0024 (11)	0.0064 (11)	0.0213 (15)
C14	0.0358 (9)	0.0635 (11)	0.0622 (11)	0.0022 (8)	0.0118 (8)	0.0231 (9)
C15	0.0438 (10)	0.0546 (10)	0.0511 (10)	−0.0077 (8)	0.0028 (8)	0.0144 (8)
C16	0.0667 (14)	0.0834 (16)	0.0601 (13)	−0.0265 (12)	0.0026 (11)	0.0038 (12)
C17	0.107 (2)	0.0806 (17)	0.0594 (13)	−0.0404 (15)	0.0067 (13)	−0.0107 (12)
C18	0.105 (2)	0.0623 (13)	0.0658 (14)	−0.0101 (13)	0.0267 (13)	−0.0163 (11)
C19	0.0612 (12)	0.0589 (12)	0.0613 (12)	0.0014 (9)	0.0152 (10)	−0.0102 (9)
N11	0.0435 (8)	0.0482 (8)	0.0525 (8)	−0.0004 (6)	0.0092 (6)	−0.0006 (7)
N20	0.0417 (8)	0.0520 (8)	0.0484 (8)	−0.0029 (6)	0.0138 (6)	0.0047 (6)
C20	0.0565 (12)	0.0711 (13)	0.0632 (12)	−0.0047 (10)	0.0224 (10)	0.0149 (10)
C21	0.0851 (17)	0.0725 (14)	0.0640 (13)	−0.0082 (12)	0.0263 (12)	0.0184 (11)
C22	0.0941 (18)	0.0619 (13)	0.0537 (12)	0.0076 (12)	0.0124 (11)	0.0127 (10)
C24	0.0443 (9)	0.0451 (9)	0.0416 (8)	−0.0004 (7)	0.0067 (7)	−0.0065 (7)
C25	0.0356 (8)	0.0490 (9)	0.0449 (9)	−0.0015 (7)	0.0074 (7)	−0.0106 (7)
C26	0.0382 (10)	0.0787 (14)	0.0643 (12)	0.0056 (9)	0.0092 (9)	−0.0058 (10)
C27	0.0356 (10)	0.0932 (17)	0.0808 (15)	−0.0046 (10)	0.0204 (10)	−0.0164 (13)
C28	0.0515 (11)	0.0793 (14)	0.0750 (14)	−0.0152 (11)	0.0347 (10)	−0.0095 (11)
C29	0.0471 (10)	0.0600 (11)	0.0604 (11)	−0.0049 (8)	0.0252 (9)	0.0006 (9)
N21	0.0356 (7)	0.0481 (8)	0.0470 (7)	−0.0026 (6)	0.0156 (6)	−0.0036 (6)
N30	0.0588 (11)	0.0760 (12)	0.0703 (11)	0.0131 (9)	0.0142 (9)	0.0083 (10)
C30	0.0503 (10)	0.0544 (11)	0.0604 (11)	0.0062 (8)	0.0114 (9)	−0.0080 (9)
C31	0.0506 (11)	0.0706 (13)	0.0729 (13)	0.0050 (10)	0.0130 (10)	−0.0043 (11)
C23	0.0614 (12)	0.0612 (12)	0.0510 (10)	0.0114 (9)	0.0057 (9)	0.0024 (9)
C32	0.0615 (14)	0.0809 (16)	0.0920 (17)	−0.0015 (12)	0.0284 (13)	−0.0054 (14)
C33	0.0883 (18)	0.0790 (16)	0.0859 (17)	0.0012 (14)	0.0393 (15)	0.0057 (13)
C34	0.0789 (17)	0.0874 (18)	0.0802 (16)	0.0168 (14)	0.0213 (13)	0.0194 (14)
N40	0.0546 (11)	0.0821 (13)	0.0871 (14)	0.0114 (9)	0.0154 (10)	0.0058 (11)
C40	0.0504 (11)	0.0597 (12)	0.0739 (13)	−0.0028 (9)	0.0149 (10)	0.0118 (10)
C41	0.0511 (12)	0.0830 (16)	0.0892 (17)	−0.0076 (11)	0.0129 (11)	−0.0039 (13)
C42	0.0705 (16)	0.101 (2)	0.0909 (19)	−0.0179 (14)	0.0202 (14)	−0.0150 (16)
C43	0.0837 (18)	0.0873 (18)	0.102 (2)	−0.0051 (14)	0.0361 (16)	−0.0132 (15)
C44	0.0668 (16)	0.095 (2)	0.106 (2)	0.0150 (14)	0.0247 (15)	−0.0003 (17)

### Geometric parameters (Å, º)

**Table:**

Mn1—N1	2.1501 (18)	C22—C23	1.381 (3)
Mn1—N2	2.1546 (17)	C22—H22	0.9300
Mn1—N21	2.2669 (13)	C24—C23	1.386 (3)
Mn1—N10	2.2746 (14)	C24—C25	1.479 (2)
Mn1—N20	2.2833 (15)	C25—N21	1.348 (2)
Mn1—N11	2.2859 (15)	C25—C26	1.389 (2)
N1—C1	1.155 (2)	C26—C27	1.372 (3)
C1—S1	1.609 (2)	C26—H26	0.9300
N2—C2	1.154 (2)	C27—C28	1.365 (3)
C2—S2	1.6203 (19)	C27—H27	0.9300
N10—C14	1.339 (3)	C28—C29	1.383 (3)
N10—C10	1.342 (3)	C28—H28	0.9300
C10—C11	1.370 (3)	C29—N21	1.335 (2)
C10—H10	0.9300	C29—H29	0.9300
C11—C12	1.362 (4)	N30—C34	1.326 (3)
C11—H11	0.9300	N30—C30	1.340 (3)
C12—C13	1.377 (4)	C30—C31	1.394 (3)
C12—H12	0.9300	C30—C30^i^	1.489 (4)
C13—C14	1.396 (3)	C31—C32	1.369 (3)
C13—H13	0.9300	C31—H31	0.9300
C14—C15	1.482 (3)	C23—H23	0.9300
C15—N11	1.348 (2)	C32—C33	1.373 (4)
C15—C16	1.384 (3)	C32—H32	0.9300
C16—C17	1.373 (4)	C33—C34	1.370 (4)
C16—H16	0.9300	C33—H33	0.9300
C17—C18	1.367 (4)	C34—H34	0.9300
C17—H17	0.9300	N40—C44	1.322 (3)
C18—C19	1.380 (3)	N40—C40	1.347 (3)
C18—H18	0.9300	C40—C41	1.384 (3)
C19—N11	1.333 (2)	C40—C40^ii^	1.479 (5)
C19—H19	0.9300	C41—C42	1.368 (4)
N20—C20	1.335 (2)	C41—H41	0.9300
N20—C24	1.343 (2)	C42—C43	1.371 (4)
C20—C21	1.373 (3)	C42—H42	0.9300
C20—H20	0.9300	C43—C44	1.380 (4)
C21—C22	1.362 (4)	C43—H43	0.9300
C21—H21	0.9300	C44—H44	0.9300
			
N1—Mn1—N2	96.69 (7)	C22—C21—H21	120.8
N1—Mn1—N21	100.33 (6)	C20—C21—H21	120.8
N2—Mn1—N21	92.88 (6)	C21—C22—C23	119.6 (2)
N1—Mn1—N10	92.04 (7)	C21—C22—H22	120.2
N2—Mn1—N10	97.38 (6)	C23—C22—H22	120.2
N21—Mn1—N10	162.91 (6)	N20—C24—C23	121.42 (18)
N1—Mn1—N20	90.37 (7)	N20—C24—C25	115.79 (15)
N2—Mn1—N20	163.68 (6)	C23—C24—C25	122.79 (17)
N21—Mn1—N20	71.33 (5)	N21—C25—C26	121.16 (18)
N10—Mn1—N20	97.05 (5)	N21—C25—C24	115.91 (14)
N1—Mn1—N11	163.00 (6)	C26—C25—C24	122.92 (17)
N2—Mn1—N11	89.80 (6)	C27—C26—C25	119.4 (2)
N21—Mn1—N11	95.00 (5)	C27—C26—H26	120.3
N10—Mn1—N11	71.49 (6)	C25—C26—H26	120.3
N20—Mn1—N11	87.59 (5)	C28—C27—C26	119.67 (19)
C1—N1—Mn1	161.31 (18)	C28—C27—H27	120.2
N1—C1—S1	179.3 (2)	C26—C27—H27	120.2
C2—N2—Mn1	175.86 (16)	C27—C28—C29	118.3 (2)
N2—C2—S2	179.25 (19)	C27—C28—H28	120.8
C14—N10—C10	118.54 (17)	C29—C28—H28	120.8
C14—N10—Mn1	117.53 (12)	N21—C29—C28	123.0 (2)
C10—N10—Mn1	123.21 (14)	N21—C29—H29	118.5
N10—C10—C11	123.5 (2)	C28—C29—H29	118.5
N10—C10—H10	118.3	C29—N21—C25	118.38 (15)
C11—C10—H10	118.3	C29—N21—Mn1	123.00 (12)
C12—C11—C10	118.1 (3)	C25—N21—Mn1	118.53 (11)
C12—C11—H11	121.0	C34—N30—C30	117.5 (2)
C10—C11—H11	121.0	N30—C30—C31	121.5 (2)
C11—C12—C13	119.9 (2)	N30—C30—C30^i^	116.7 (2)
C11—C12—H12	120.0	C31—C30—C30^i^	121.8 (2)
C13—C12—H12	120.0	C32—C31—C30	119.3 (2)
C12—C13—C14	119.2 (3)	C32—C31—H31	120.4
C12—C13—H13	120.4	C30—C31—H31	120.4
C14—C13—H13	120.4	C22—C23—C24	119.0 (2)
N10—C14—C13	120.7 (2)	C22—C23—H23	120.5
N10—C14—C15	116.21 (15)	C24—C23—H23	120.5
C13—C14—C15	123.0 (2)	C31—C32—C33	119.4 (2)
N11—C15—C16	120.7 (2)	C31—C32—H32	120.3
N11—C15—C14	115.93 (16)	C33—C32—H32	120.3
C16—C15—C14	123.36 (19)	C34—C33—C32	117.6 (2)
C17—C16—C15	119.3 (2)	C34—C33—H33	121.2
C17—C16—H16	120.3	C32—C33—H33	121.2
C15—C16—H16	120.3	N30—C34—C33	124.7 (2)
C18—C17—C16	120.3 (2)	N30—C34—H34	117.7
C18—C17—H17	119.9	C33—C34—H34	117.7
C16—C17—H17	119.9	C44—N40—C40	117.4 (2)
C17—C18—C19	117.6 (2)	N40—C40—C41	121.3 (2)
C17—C18—H18	121.2	N40—C40—C40^ii^	116.7 (2)
C19—C18—H18	121.2	C41—C40—C40^ii^	122.0 (3)
N11—C19—C18	123.2 (2)	C42—C41—C40	119.8 (2)
N11—C19—H19	118.4	C42—C41—H41	120.1
C18—C19—H19	118.4	C40—C41—H41	120.1
C19—N11—C15	118.94 (17)	C41—C42—C43	119.5 (3)
C19—N11—Mn1	123.75 (13)	C41—C42—H42	120.3
C15—N11—Mn1	117.23 (13)	C43—C42—H42	120.3
C20—N20—C24	118.28 (17)	C42—C43—C44	117.2 (3)
C20—N20—Mn1	123.43 (13)	C42—C43—H43	121.4
C24—N20—Mn1	118.28 (11)	C44—C43—H43	121.4
N20—C20—C21	123.3 (2)	N40—C44—C43	124.8 (3)
N20—C20—H20	118.3	N40—C44—H44	117.6
C21—C20—H20	118.3	C43—C44—H44	117.6
C22—C21—C20	118.4 (2)		

Symmetry codes: (i) −*x*+1, −*y*+1, −*z*+1; (ii) −*x*+2, −*y*+1, −*z*+1.
