# Supplementary figures and images for: Crystal structure of bis­(2,2′-bi­pyridine-κ2 N,N′)bis­(thio­cyanato-κN)mang­anese(II) 2,2′-bi­pyridine monosolvate
Source: Acta Crystallogr E Crystallogr Commun. 2015 Jan 1;71(Pt 1):m3–4. doi: 10.1107/S205698901402516X (PMC4331911; doi:10.1107/S205698901402516X)

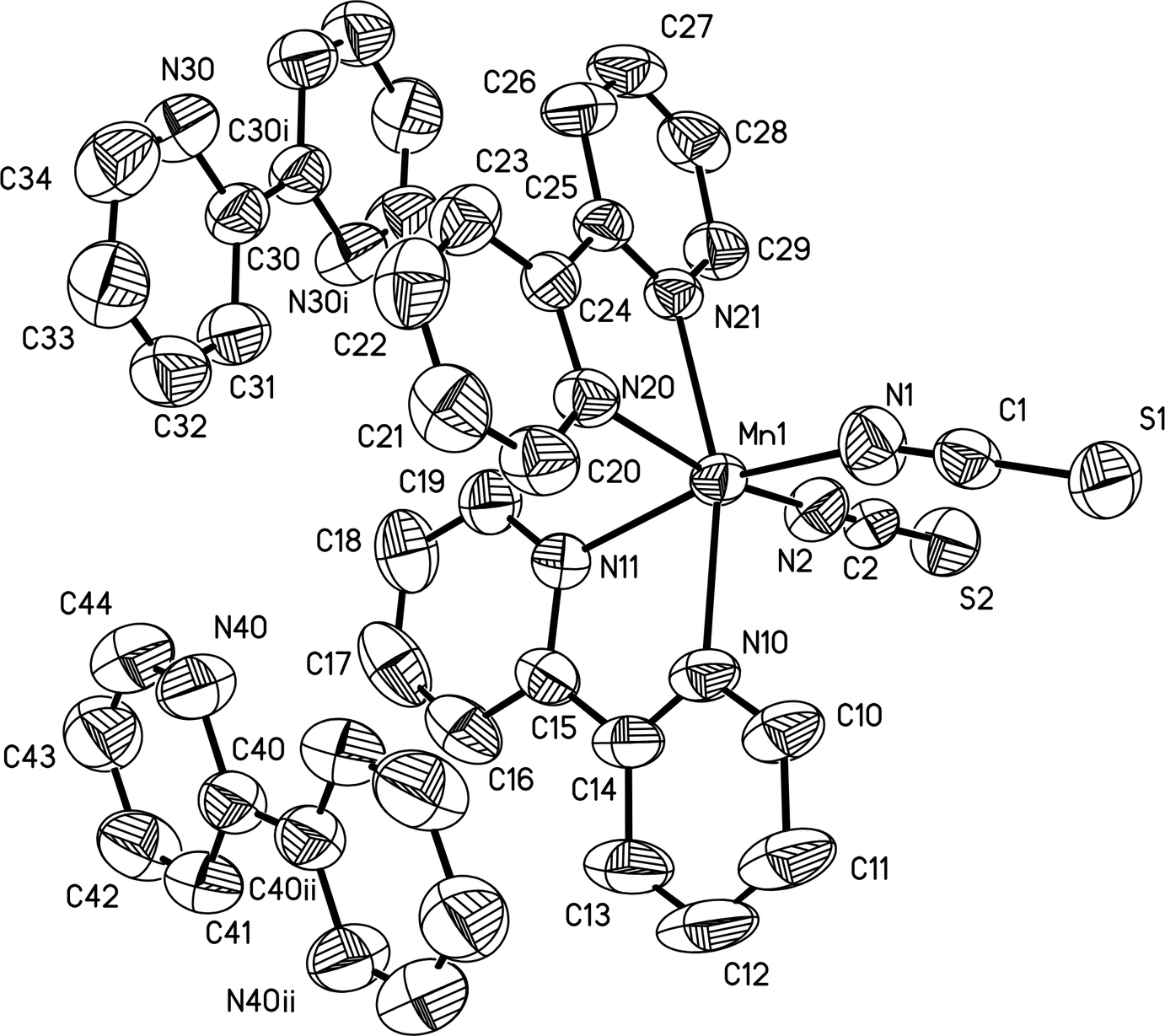

Supplement: Supplementary file 3 [file e-71-000m3-fig1.tif]

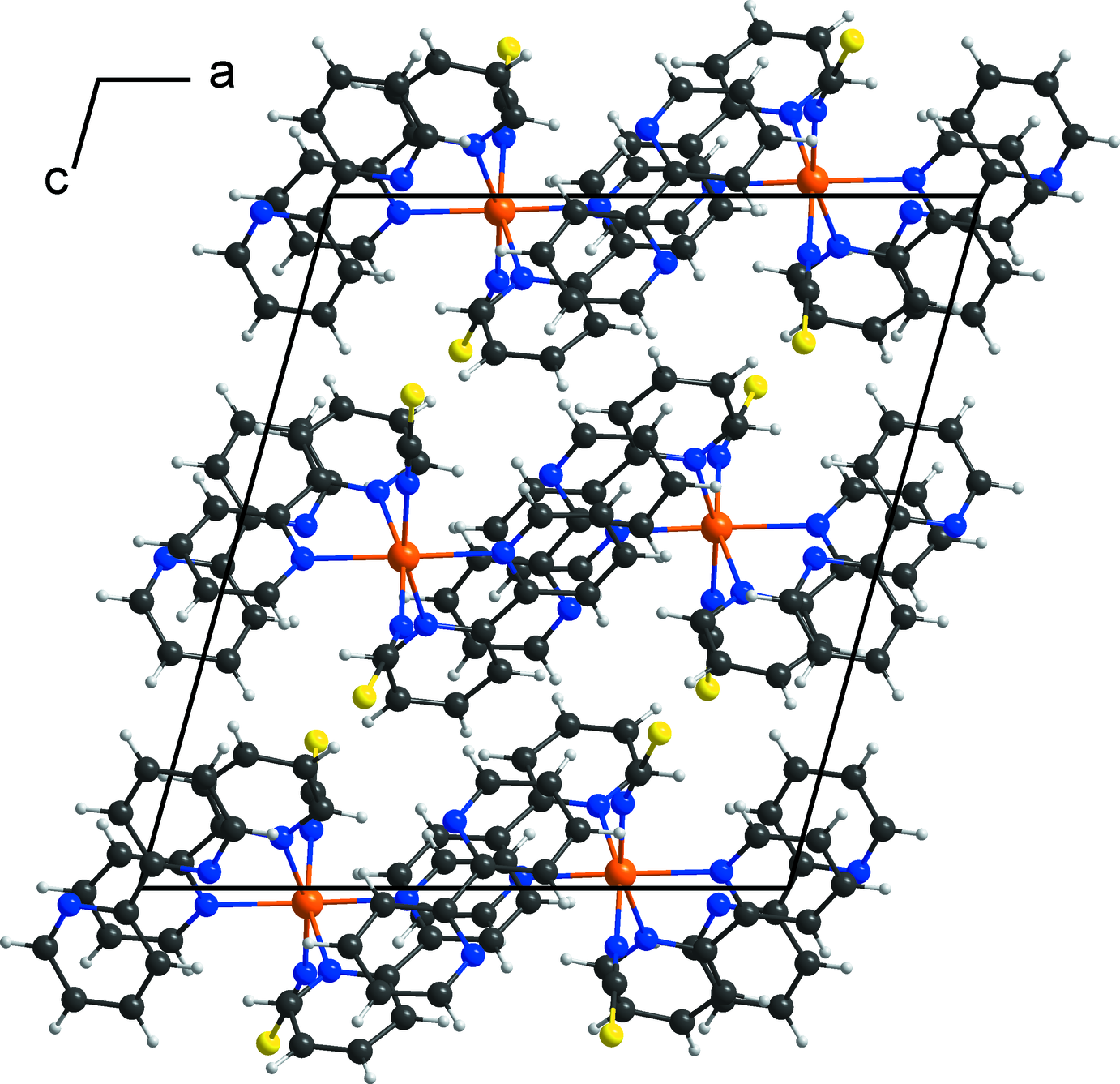

Supplement: Supplementary file 4 [file e-71-000m3-fig2.tif]
